# Acquired Generalized Lipodystrophy Associated with Autoimmune Hepatitis and Low Serum C4 Level

**DOI:** 10.4274/jcrpe.v2i1.39

**Published:** 2010-12-08

**Authors:** Erdal Eren, Tanju Başarır Özkan, Esra D. Papatya Çakır, Halil Sağlam, Ömer Tarım

**Affiliations:** 1 Department of Pediatric Endocrinology, Uludağ University Faculty of Medicine, Bursa, Turkey; 2 Department of Pediatric Gastroenterology, Uludağ University Faculty of Medicine, Bursa, Turkey; +90 224 295 05 40+90 224 442 81 43erderen@yahoo.comErdal Eren, Uludağ University, Medical Faculty Department of Pediatric Endocrinology, Bursa, Turkey

**Keywords:** Lipodystrophy, autoimmune hepatitis, complement C4

## Abstract

Lipodystrophies are a group of diseases characterized by loss of fat tissue and are associated with insulin resistance. A six-year- old girl followed with the diagnosis of autoimmune hepatitis showed a severe loss of fat tissue, hyperinsulinemia, impaired glucose tolerance, hypertriglyceridemia and low serum complement 4 (C4) levels. She had coarse facial features with generalized loss of subcutaneous fat and prominent muscularity. Remarkable acanthosis nigricans was present over the neck, axilla, and umbilicus. Two hours after glucose loading, the glucose tolerance test revealed a glucose level of 258 mg/dL, a HbA1c value of 6.8%, and an insulin level of 642.9 mIU/mL, documenting a state of insulin resistance and type 2 diabetes mellitus. Acquired generalized lipodystrophy was diagnosed and metformin with dietary intervention was initiated. Low serum complement levels proved the autoimmune nature of the process. We conclude that the serum complement levels must be investigated in patients with acquired lipodystrophy, particularly when it is associated with autoimmune hepatitis.

**Conflict of interest:**None declared.

## INTRODUCTION

Lipodystrophies are rare diseases characterized by loss of fat tissue in the body. This group of diseases may be congenital or acquired, and each has several subtypes which may be generalized or local. The congenital generalized form is also known as Berardinelli-Seip syndrome (1). Hyperinsulinemia, insulin resistance, hyperglycemia, hypertriglyceridemia, and fatty liver are other features of this syndrome. The pathogenesis of congenital generalized lipodystrophy is not clear. Fat tissue has endocrine, paracrine, and autocrine effects in addition to its role in energy storage (2). The components of the classical complement pathway are also synthesized in fat tissue (3). Consequently, it has been proposed that complement activation may be the cause of lipodystrophy (4, 5, 6, 7). Recently, three cases with autoimmune hepatitis and acquired lipodystrophy with low complement 4 (C4) levels have been reported (8). This paper presents a case with autoimmune hepatitis who developed generalized lipodystrophy.

## CASE REPORT

A six-year-old girl was admitted to the hospital with abdominal distention, respiratory distress, and hyperglycemia. She had been followed by the departments of gastroenterology and cardiology with diagnoses of autoimmune hepatitis and hypertrophic cardiomyopathy.

A liver biopsy was performed at age one and a half years because of hypertransaminasemia (aspartate transaminase [AST] 379, alanin transaminase [ALT] 546 U/L) and was reported as chronic hepatitis. At that time, total bilirubin level was 1.1 mg/dL and direct bilirubin level was 0.7 mg/dL. Serum triglyceride level was elevated (496 mg/dL). Six months later (at the age of two years), the patient was readmitted with fever and haematuria. Her liver was 6 cm and spleen 2 cm palpable below the costal margin. The laboratory evaluation at that time revealed elevated transaminases (AST 152 and ALT 166 U/L), positive antimitochondrial antibodies (AMA) and anti-liver-kidney microsome antibodies (LKM1). Nephrocalcinosis was reported on ultrasonographic examination. 

The patient was born to a sixteen-year-old mother by vaginal delivery at full term and her weight was 2250 g. The parents reported that her appearance was normal during the first year of her life. Subsequently, they had noted that she appeared thinner with reduced subcutaneous tissue. There was no family history of consanguinity and her three-year-old sister was healthy.

**Figure 1:**
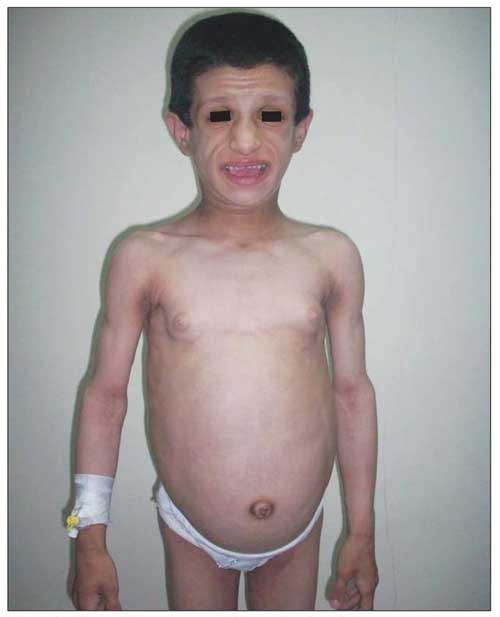
General appearance of the patient, note the coarse face, generalized loss of subcutaneous fat, prominent muscularity, and protuberant abdomen

**Figure 2:**
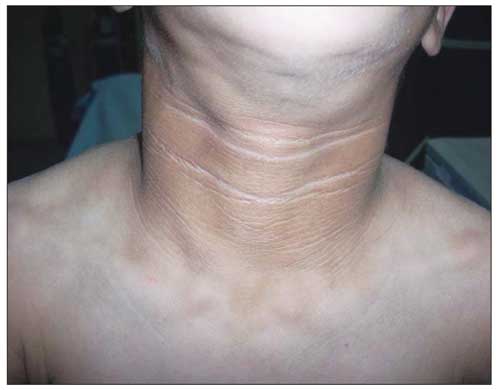
Remarkable acanthosis nigricans over the neck

**Figure 3:**
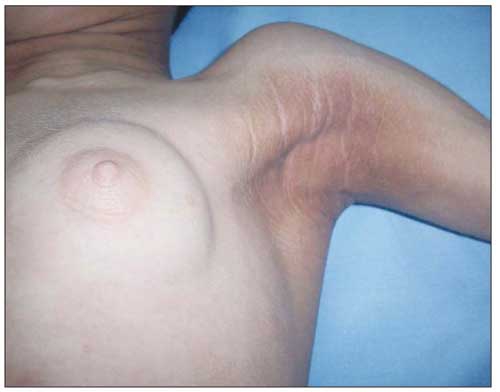
Note the acanthosis nigricans on the axilla and pseudothelarche

On physical examination, the patient’s weight (23 kg) and height (117 cm) were above the 97th percentile. Her weight for height was normal. She was mentally dull. She had coarse facial features with generalized loss of subcutaneous fat and prominent muscularity (Figure 1). Her tonsils were hypertrophic. Remarkable acanthosis nigricans was present over the neck, axilla, and umbilicus (Figure 2). The abdomen was protuberant and distended with hepatosplenomegaly. The liver was palpable 6 cm below the costal margin and the spleen was massively enlarged, extending to the inguinal area. Dyspnea with subcostal retractions was present and coarse crackles were audible over the entire chest. There was a systolic murmur of 2-3/6 magnitude over the mesocardiac area. Her pubertal status was Tanner stage III for thelarche (pseudothelarche) and stage I for pubarche (Figure 3).

The patient had lipomastia that simulated breast enlargement corresponding to a Tanner stage III thelarche, although the tissue was lipoid rather than glandular. This was confirmed by prepubertal gonadotropin and estradiol levels. The bone age was also appropriate for chronological age.

The laboratory results were as follows: Hb 12.8 g/dL, MCV 86 fl, platelet count 131.000/mm^3^, WBC 10 140/mm^3^, glucose 185 mg/dL, ALT 88 U/L, AST 110 U/L, total bilirubin 1.9 mg/dL, direct bilirubin 0.5 mg/dL, triglyceride 438 mg/dL, total cholesterol 158 mg/dL, LDL 20 mg/dL, and HDL 20 mg/dL. Anti-smooth muscle antibody (ASMA) and anti-nuclear antibody (ANA) both were positive at 1/100 dilution. Two hours after a glucose load of 1.75 g/kg blood glucose level was 258 mg/dL, HbA1c 6.8% and insulin level was 642.9 mIU/mL, revealing a state of insulin resistance and type 2 diabetes mellitus (DM). 

Serum adiponectin (<0.3 mcg/mL) and leptin (0.1 mcg/L) levels were both below the detectable range. Serum complement 3 (C3) level was normal (1.11 g/L [0.8-2.14]), but serum C4 level was low (0.1 g/L [0.13-0.6]). Immune globulin G (IgG) was elevated at 24.2 g/L (4.9-16.1). Anti-LKM was positive. 

Acquired generalized lipodystrophy was diagnosed based on these findings. Metformin therapy was started at a dose of 850 mg bid orally along with dietary intervention. Although the patient did not attend the follow-up visits regularly, during her last visit it was noted that acanthosis nigricans decreased tremendously. One year after starting metformin and diet therapy, her fasting blood glucose and insulin levels were 123 mg/dL and 85.2 mIU/mL, respectively. HbA1c was 6.2%. Recently, corticosteroid therapy was started for autoimmune hepatitis. Currently, the patient is still being followed for cardiomyopathy, autoimmune hepatitis, type 2 diabetes, and insulin resistance. 

The parents provided verbal and written permission for taking and publishing photographs of the patient.

## DISCUSSION

The risk of type 2 DM with increasing fat and insulin resistance is well-known (9). On the other hand, loss of fat tissue may also be associated with insulin resistance and type 2 DM. It has been suggested that insulin resistance in lipodystrophy may be associated with accumulation of ectopic fat tissue in the liver, in skeletal muscles, and in pancreatic beta cells (10). The diminished fat tissue may lead to increased fatty acids, which may be stored as triglycerides in the liver and muscles. In the long term, the increased free fatty acids lead to a reduced beta-cell response to hyperglycemia. In congruence with the loss of fat tissue, leptin and adiponectin levels decrease to undetectable levels (8). As we have observed in our patient, serum adiponectin and leptin levels that reveal the amount of fat in the body are decreased in this condition. It has also been reported that resistin level is reduced and tumor necrosis factor (TNF)-a which is a proinflammatory cytokine is increased (11). Recently, leptin therapy has been reported to correct hyperglycemia, dyslipidemia, and hepatic steatosis both in congenital and acquired forms of lipodystrophy (12, 13, 14, 15). Unfortunately, leptin therapy was not available for our patient. 

The pathogenesis of acquired generalized lipodystrophy is not completely understood at present. Many patients with congenital lipodystrophy have low C3 levels associated with mesangiocapillary glomerulonephritis (7). This possibility could have explained the haematuria and proteinuria in our patient. Serum C3 concentration is decreased by activation of the alternate pathway. Acquired lipodystrophy frequently coexists with other autoimmune diseases. Severe hemolytic anemia, autoimmune thyroid disease, and polyneuropathy may be present. Although rare, neurological problems have been reported (16). Although nonalcoholic fatty liver is seen in all patients with generalized lipodystrophy, in some patients, fatty liver has been reported to occur before lipodystrophy is evident (17). The hypertrophic cardiomyopathy in our patient was an expected finding associated with hyperinsulinemia. 

Ziegler (18) reported acquired lipodystrophy for the first time in 1928. The first patient with acquired lipodystrophy who presented with steatohepatitis was reported in 1989 (19). 

Serum C4 of the present case was decreased. Savage et al (8) have reported the association of diminished C4 and autoimmune hepatitis in three patients with generalized lipodystrophy and proposed that low C4 might be the causative factor in this disease. On the other hand, low C4 is also a well-known finding in patients with autoimmune hepatitis (20, 21). It has been reported that children with autoimmune hepatitis have an isolated partial deficiency of the complement component C4, which is genetically determined and is associated with the possession of the silent gene C4AQO at the C4A locus (22, 23, 24). Therefore, low C4, which is a feature of autoimmune diseases, could be genetically determined and could have resulted in both autoimmune hepatitis and lipodystrophy, if it is the causative factor. Unfortunately, detailed genetic analysis was not available for this case. It appears that further genetic studies can be performed to enlighten the possible autoimmune basis of acquired lipodystrophies. 

In conclusion, our patient provides another example for acquired generalized lipodystrophy in which autoimmune hepatitis was the initial manifestation. Since autoimmune hepatitis may precede lipodystrophy, this entity must be kept in mind as a diagnostic possibility. We do not know yet whether low C4, which may be inherent, is a triggering factor for both autoimmune hepatitis and acquired lipodystrophy, or not.

## ACKNOWLEDGEMENT

The authors thank Dr. Robert Semple from University of Cambridge, Department of Clinical Biochemistry, Metabolic Research Laboratories for measuring serum leptin, adiponectin, and C4 levels.
